# How to Use SNP_TATA_Comparator to Find a Significant Change in Gene Expression Caused by the Regulatory SNP of This Gene's Promoter via a Change in Affinity of the TATA-Binding Protein for This Promoter

**DOI:** 10.1155/2015/359835

**Published:** 2015-10-04

**Authors:** Mikhail Ponomarenko, Dmitry Rasskazov, Olga Arkova, Petr Ponomarenko, Valentin Suslov, Ludmila Savinkova, Nikolay Kolchanov

**Affiliations:** ^1^Institute of Cytology and Genetics, Siberian Branch of Russian Academy of Sciences, Novosibirsk 630090, Russia; ^2^Department of Natural Sciences, Novosibirsk State University, Novosibirsk 630090, Russia; ^3^Children's Hospital Los Angeles, University of Southern California, Los Angeles, CA 90027, USA

## Abstract

The use of biomedical SNP markers of diseases can improve effectiveness of treatment. Genotyping of patients with subsequent searching for SNPs more frequent than in norm is the only commonly accepted method for identification of SNP markers within the framework of translational research. The bioinformatics applications aimed at millions of unannotated SNPs of the “1000 Genomes” can make this search for SNP markers more focused and less expensive. We used our Web service involving Fisher's *Z*-score for candidate SNP markers to find a significant change in a gene's expression. Here we analyzed the change caused by SNPs in the gene's promoter via a change in affinity of the TATA-binding protein for this promoter. We provide examples and discuss how to use this bioinformatics application in the course of practical analysis of unannotated SNPs from the “1000 Genomes” project. Using known biomedical SNP markers, we identified 17 novel candidate SNP markers nearby: rs549858786 (rheumatoid arthritis); rs72661131 (cardiovascular events in rheumatoid arthritis); rs562962093 (stroke); rs563558831 (cyclophosphamide bioactivation); rs55878706 (malaria resistance, leukopenia), rs572527200 (asthma, systemic sclerosis, and psoriasis), rs371045754 (hemophilia B), rs587745372 (cardiovascular events); rs372329931, rs200209906, rs367732974, and rs549591993 (all four: cancer); rs17231520 and rs569033466 (both: atherosclerosis); rs63750953, rs281864525, and rs34166473 (all three: malaria resistance, thalassemia).

## 1. Introduction

Biomedical SNP (single nucleotide polymorphism) markers are significantly frequent differences of personal genomes of patients from the reference human genome, hg19. The discovery of SNP markers of hypersensitivity to the HIV-1 reverse transcriptase inhibitor Ziagen in the* HLA-B* gene of the human major histocompatibility complex [1] prevented deaths of thousands of patients. That is the reason why a search for candidate SNP markers of diseases now represents the bulk of bioinformatics studies aimed at the development of so-called postgenomic predictive preventive personalized medicine, PPPM [2].

In the 20th century, discovery of SNPs and of the resulting associations with diseases was casual, whereas the postgenomic search for SNPs is systematic and large-scale: it includes the largest worldwide project “1000 Genomes” [3]. Researchers maintaining the dbSNP database [4] accumulate and annotate proven SNPs and continuously refine the human reference genome (hg19), namely, the ancestral variants for all SNPs within the Ensembl [5] and GENCODE v. 19 [6] databases available from the public UCSC Genome Browser [7]. The biomedical databases GWAS (genome-wide association study) [8], OMIM [9], ClinVar [10], and HapMap [11] supplement these SNPs by documenting associations with diseases, with one another, and with the pathogenic haplotypes (e.g., [12]). Furthermore, researchers project these SNPs onto the whole-genome maps of genes, protein-binding sites on DNA predicted* in silico* and/or detected* in vivo* using chromatin immunoprecipitation (ChIP), interchromosomal contacts, and nucleosome packaging as well as transcriptomes in health [13] and disease in different tissues [14] and after treatment [15]. Accordingly, the available Web services (e.g., [16–27]) facilitate the bioinformatics search for relevant-to-medicine candidate SNP markers in terms of ranking of unannotated SNPs by their similarity to known biomedical SNP markers, according to projections of these SNPs onto the whole-genome maps. The Central Limit Theorem means [28] that the accuracy of such a search should increase asymptotically with an increase in accuracy, volume, representativeness, completeness, the number, and diversity of the whole-genome maps as well as due to refinement of empirical analyses of similarity between projections of SNPs onto genomic maps [16]. This way, the best research progress has been achieved for many thousands of SNPs within protein-coding regions of genes [9] due to the invariant types of disruption in both structure and function of the affected proteins regardless of the cellular conditions [29]. At the same time, the worst research progress has been made for a few hundred of so-called regulatory SNPs [4, 9, 23, 24] because their manifestations are dependent on cellular conditions [30].

For the present study, it was helpful that an intermediate position between these extremes belongs to SNPs in the DNA sites binding to the TATA-binding protein (TBP); these SNPs constitute ~10% of all the known regulatory SNP markers relevant to medicine, whereas TBP is only one of 2600 known DNA-binding proteins in humans [31]. The above-mentioned special place of such SNPs can be mostly explained by the necessity of a TBP-binding site within the [−70; −20] region of the promoter for any mRNA [32] because RNA polymerase II binds to the anchoring complex TBP-promoter, and this event triggers assembly of the transcription preinitiation complex for this mRNA [33]. These results were obtained in studies on unviability of* TBP*-null animals [34] or animals harboring a knockdown [35] of the* TBP* gene. Besides, ChIP data confirmed that the ТАТА-like motifs are the TBP-binding sites in gene promoters in yeast [36] and in mice [37], as did the results of* in silico* analysis and their selective verification by means of* in vivo* bioluminescence among human genes [38]. Finally, SNPs in the TBP-binding sites invariantly cause gene overexpression in relation to SNP-caused enhancement of the TBP/promoter affinity as well as the deficient expression of genes as a result of an SNP-caused reduction in this affinity regardless of any cellular conditions; these phenomena have been repeatedly demonstrated in independent experiments [39–41]. This stability of the SNP-caused alterations in the TBP/promoter-affinity resembles the invariant relation of SNPs in protein-coding gene regions with protein structure/function, rather than such relations involving regulatory SNPs, whose effects strongly depend on the tissue, cell type, and so forth.

In our previous studies, we measured* in vitro* affinity values of TBP for the representative sets of aptamers of synthetic single-stranded DNA (ssDNA) [42] and double-stranded DNA (dsDNA) [43] including natural TBP-binding sites of human gene promoters [44] that are stored in our database ACTIVITY [45]. Next, we derived formulas for* in silico* prognosis of the TBP-ssDNA [46], TBP-dsDNA [43], and TBP-promoter [47] affinity using the widely accepted Bucher's criterion [48] for the canonical TBP-binding sites, the so-called TATA box (synonyms: Goldberg-Hogness box and Hogness box [32]), in the three-step mechanism of the TBP binding to a promoter [47]. This mechanism was observed independently* in vitro* a year later [49]. Then we confirmed predictions of this three-step empirical predictive bioinformatics model [47] at equilibrium [50], without equilibrium [51], and in real time [52, 53]* in vitro*. Additionally, we compiled a set of SNPs in the TBP-binding sites associated with human diseases [54], including the AIDS pandemic [55], and with commercially important traits of plants and animals [56]. Then, we confirmed the three-step predictions by means of these SNPs [57] and by means of transcriptomes of the human brain [58], the auxin response in plants [59, 60], and the data from 68 independent experiments (for review, see [61]). To finalize this comprehensive verification of the three-step model of TBP binding to a promoter [47, 49], we created a freely available Web service [62] for users who wish to apply this bioinformatics application to data on the TBP/promoter-complexes in humans: http://beehive.bionet.nsc.ru/cgi-bin/mgs/tatascan/start.pl.

In this work, we updated our review of SNPs (in the TBP-binding sites) associated with human diseases [54] using the standard keyword search, using existing data from the literature [63], in NCBI databases [4] and provide examples on how to use our Web service [62] to find a significant change in a gene's expression when this change is caused by the regulatory SNP in this gene's promoter* via* a change in the TBP affinity for the promoter. Using a representative set of so-called control data on the total number of 62 SNPs, we show the output of our bioinformatics applications. Using this approach, for the known SNP markers relevant to medicine, we present 17 novel candidate SNP markers that are located nearby, namely, rs549858786 of the* IL1B* gene (associated with rheumatoid arthritis), rs63750953 and rs281864525 (both:* HBB*; malaria resistance and *β*-thalassemia), rs34166473 (*HBD*; malaria resistance and *δ*-thalassemia), rs563558831 (*CYP2B6*; better bioactivation of cyclophosphamide), rs372329931 (*ADH7*; esophageal cancer), rs562962093 (*MBL2*; stroke, preeclampsia, and variable immunodeficiency), rs72661131 (*MBL2*; cardiovascular events in rheumatoid arthritis), rs17231520 and rs569033466 (both:* CETP*; atherosclerosis), rs55878706 (*DARC*; low white-blood-cell count and resistance to malaria), rs367732974 and rs549591993 (both:* F7*; progression of colorectal cancer from a primary tumor to metastasis), rs572527200 (*MMP12*; low risks of asthma, systemic sclerosis, and psoriasis), rs371045754 (*F9*; Leiden hemophilia B), rs200209906 (*GSTM3*; brain, lung, and testicular cancers), and rs587745372 (*GJA5*, arrhythmia and cardiovascular events). This is the principal result of this work.

## 2. Methods

### 2.1. Web-Service SNP_TATA_Comparator

Web service SNP_TATA_Comparator http://beehive.bionet.nsc.ru/cgi-bin/mgs/tatascan/start.pl [62] is a bioinformatics application installed on the hybrid cluster supercomputer HKC-30T (Hewlett Packard, Palo Alto, CA, US) based on the Intel Xeon 5450 platform of 85-Tflop performance under OS Red Hat Enterprise Linux 5.4 that is supported by the Siberian Supercomputer Center (Novosibirsk, Russia).

**Figure 1:**
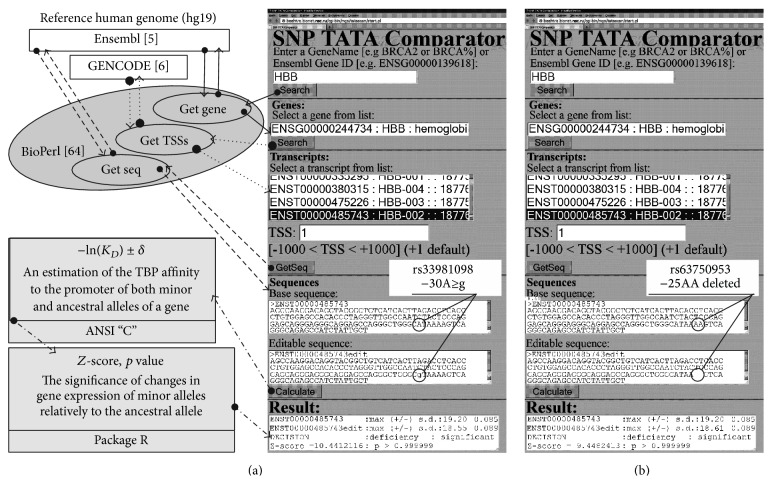
How to use the Web service SNP_TATA_Comparator [62] to find a significant change in gene expression caused by SNPs of this gene's promoter via a change in affinity of the TATA-binding protein (TBP) for this promoter in the cases of (a) a known biomedical SNP marker and (b) a nearby candidate SNP marker. Solid, dotted, and dashed arrows are the gene, transcript, and sequence lists, respectively, from Ensembl [5] and GENCODE [6] databases of the reference human genome, hg19. Dash-and-dot arrows are an estimate of the statistical significance (*Z*-score, *p* value) of deviation of the gene expression in patients carrying minor alleles, relative to the ancestral allele, (1)–(4) and Algorithm 1.

One can see screenshots of the user interface of this software in Figure 1 and all the data flowcharts (arrows) between them and two databases Ensembl [5] and GENCODE v. 19 [6] of the human reference genome, hg19, in Figure 1(a). Using the standard method, we encoded this interface in the dynamic programming language JavaScript and created these flowcharts by means of the BioPerl toolkit [64]. Using the online mode of these modules, a user can prepare input data for the executable applet encoded primarily in the programming language C of the ANSI standard and, then, run this applet (the “Calculate” button). These input data consist of two variants—ancestral (the “Base sequence” window) and minor (the “Editable sequence” window)—of the 90 bp DNA sequence {*s*_−90_ ⋯ *s*_*i*_ ⋯ *s*_−1_} in the proximal core-promoter region immediately upstream of the transcription start site (TSS, *s*_0_) of interest within the human reference genome, hg19 (where *s*_*i*_ ∈ {*a*, *c*, *g*, *t*}). One can find our description of the bioinformatics model of this executable applet within the next Section 2.2.

One more example of the output data from the above-mentioned executable applet is shown within the two top lines of the “Result” window in Figure 1(b). These data include the maximum value, −ln⁡(*K*_*D*_) ± *δ*, among all the possible estimates of the TBP binding affinity for the 26 bp DNA fragment, {*s*_*i*−13_ ⋯ *s*_*i*_ ⋯ *s*_*i*+12_} at the *i*th position ranging from –70 to –20 for both DNA chains [32, 59]. Here, *K*_*D*_ is the equilibrium dissociation constant (expressed in the units of mol per liter; M) of the TBP binding to the ancestral or minor allele of the promoter under study. These quantitative estimates of the SNP-caused change in the TBP-promoter affinity are the input data for another executable applet coded primarily by means of the standard statistical package in the R software. We provided examples of its output data within the bottom line of the “Result” window in Figure 1. These are Fisher's *Z*-score value along with its probability rate, *p* (where *α* = 1 − *p*, statistical significance). Within the “Decision” line, one can see the prediction made by our Web service, namely, (i) “excess” for overexpression of the gene after the SNP-caused significant increase in the TBP binding affinity for the minor allele of the gene promoter or (ii) “deficiency” for lowered expression of this gene in the opposite case. This prediction is the main result of the proposed Web service [62].

### 2.2. The Bioinformatics Model

The bioinformatics model that we use here is the three-step approximation of the TBP binding to the [−70; −20] region of the core-promoters of eukaryotic genes; this approximation was first suggested by us [47] on the basis of our original experimental data [42–44] and, then, this three-step approximation was discovered independently [49] a year later. Within the framework of this model, (i) TBP binds nonspecifically to DNA and slides along this molecule ↔ (ii) the sliding of TBP stops at a proper TBP-binding site ↔ the DNA helix bends from the 19° angle to the 90° angle [65] and stabilizes the local TBP-promoter complex. This interaction (binding affinity) can be estimated using the following empirical equation:

(1) −ln⁡KD=10.9−0.2ln⁡KSLIDE+ln⁡KSTOP+ln⁡KBEND,

where 10.9 (ln units) is nonspecific TBP-DNA affinity 10^−5 ^M [66], 0.2 is the stoichiometric coefficient [47], and *K*_STOP_ is the maximal score value of Bucher's position-weight matrix, which is the commonly accepted criterion of the TATA box: the canonical form of the TBP-binding site [48].

In (1), *K*_SLIDE_ is our empirical estimate of the equilibrium constant of the TBP sliding along DNA that was determined experimentally [67]; namely,

(2) −ln⁡KSLIDE=MEAN15 bp0.8TA3′HALF−3.4 MinorGrooveWidthCENTER−35.1,

where [TA]_3′HALF_ is the total number of instances of dinucleotide TA within the 3′-half of the DNA sequence treated; MinorGrooveWidth_REGION_ is the mean width of the minor groove of the B-form of the DNA helix [68]; 0.8, −3.4, and −35.1 are linear regression coefficients determined by means of our experimental data [43] stored in our database ACTIVITY [45]; MEAN_15 bp_ is the mean arithmetic value for all possible positions and orientations of the TBP-binding site (15 bp long) that was determined empirically [67].

In (1), *K*_BEND_ is our empirical estimate of the equilibrium constant at the DNA helix bending step on the basis of the macromolecular dynamics computations [65] describing how TBP can bind to DNA; namely,

(3) −ln⁡KBEND=MEANTATA-box0.9WRFLANK+2.5TVCENTER+14.4,

where WR = {TA, AA, TG, AG} and TV = {TA, TC, TG} [46] (the IUPAC-IUB nomenclature [69]); 0.9, 2.5, and 14.4 are linear regression coefficients calculated from our experimental data [42] stored in our database ACTIVITY [45]; MEAN_TATA-box_ is the mean arithmetic value for both DNA strands of the TBP-binding site at the position of the maximal score value of Bucher's position-weight matrix [48].

Additionally, the standard deviation of the −ln⁡[*K*_*D*_] estimates (see (1))—for all the 78 possible mononucleotide substitutions, *s*_*i*+*j*_ → *ξ*, at each *j*th position (−13 ≤ *j* ≤ 12; 3 × 26) within the 26 bp DNA window centered by *i*th position of the promoter DNA analyzed—was heuristically estimated as 
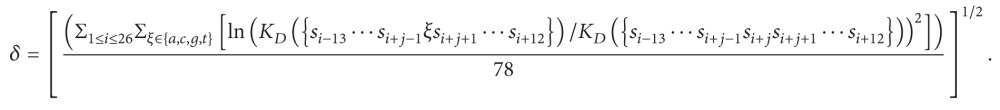
(4)


This equation (4) estimates the resistance against the majority of SNPs in the case of the biologically essential complex of TBP binding to the TBP-binding site of the promoters [55].

Finally, the results of (1)–(4) on the promoter DNA sequences of two minor and ancestral alleles of a given gene are compared with one another in terms of Fisher's *Z*-score and its probability rate, that is, the *p* value (where *α* = 1 − *p* is the statistical significance level). On this basis, a decision is made.

For each SNP processed, the decision (Algorithm 1) is the main result of the bioinformatics model used.

### 2.3. How to Use SNP_TATA_Comparator

Practical use of our Web service [62] is illustrated in Figure 1 and documented in Tables 1–3. In this work, we analyzed* in silico* 31 human genes containing 40 known biomedical SNP markers in their core-promoter from our review [54], which was updated in the present work. Using the UCSC Genome Browser [7], we found 163 additional unannotated SNPs nearby that were detected in the “1000 Genomes” project [3]. Thus, the total number of the DNA sequences processed was 203.

We used the ancestral variants of these SNPs from Ensembl [5] using the GENCODE v. 19 [6]; we also constructed their minor alleles by hand in “online real-time” mode according to the dbSNP entries [4] and/or literature sources in the case of the SNPs undocumented in this database as shown in Figure 1 and in Tables 1–3. We analyzed each of the 203 SNPs independently from one another. As a result, for most of the unannotated SNPs analyzed, we found insignificant changes in TBP affinity for human promoters: 142 of 163 or 90% of SNPs (data not shown).

Finally, the remaining 17 of the 163 unannotated SNPs (10%) appeared to be new candidate biomedical SNP markers near the existing markers. We* italicized* and labeled them with the marks “*hypothetical*” and “*this work*” in Tables 1–3. We found associations of both known and possible nearby SNP markers with the same human diseases in the case of their codirectional effects on gene expression; otherwise, we did an additional keyword search [54, 63] in NCBI databases [4] and recorded the results below the above-mentioned marks “*hypothetical*” and “*this work*.” These 17 new candidate biomedical SNP markers are the main result of the present study on how to use the proposed Web service [62] in practice.

## 3. Results

### 3.1. The Results on Seven Known Biomedical SNP Markers That Increase TBP Affinity for Human Gene Promoters

The results on seven known biomedical SNP markers that increase TBP affinity for human gene promoters are presented in Table 1. The most widely studied among them is rs1143627, a substitution of minor T for ancestral C at position −31 (hereafter denoted as −31C→T) in the core-promoter for transcript number 2 of the human* IL1B* gene (interleukin 1*β*). Let us analyze it in detail so that we can later briefly describe the rest of our SNPs on the basis of this example.

As one can see in Table 1, this SNP transforms a noncanonical TBP-binding site to the canonical TATA-box, namely, gaaagC_−31_ATAAAAcag → gaaagT_−31_ATAAAAcag. Obviously, the minor allele −31T can significantly increase TBP affinity for the* IL1B* promoter relative to the ancestral one, −31C. According to (1)–(4) and Algorithm 1, their estimate *K*_*D*_ = 2 nM (Table 1), in the case of −31T, is significantly greater (*Z*-score = 14.56, *α* < 10^−6^) than *K*_*D*_ = 5 nM in case of −31С. According to three independent empirical studies [39–41], this significant increase in TBP affinity for the minor variant of the* IL1B* promoter corresponds to overexpression of this gene (designated as ↑ in Tables 1–3). This prediction is consistent with clinical findings: overexpression of interleukin 1*β* in gastric cancer with* Helicobacter pylori* infection [10, 70], in hepatocellular carcinoma with infection by hepatitis C virus [71], in non-small cell lung cancer in smokers and during alcohol dependence [72], as well as in nonneoplastic chronic gastritis and gastric ulcer [73], in intractable Graves' autoimmune disease [74], and even in a neurodegenerative disorder during major recurrent depression [75]. Thus, the prediction by the Web service [62] (see (1)–(4) and Algorithm 1) is consistent with a number of independent clinical studies [70–75].

Using the UCSC Genome Browser [7], we found the unannotated SNP rs549858786 (−28A→T) positioned 4 bp downstream of the above-mentioned known SNP marker rs1143627 (–31C→T). As one can see in Figure 1(b), our Web service [63] predicts (see (1)–(4) and Algorithm 1) the affinity of TBP for the minor allele −28T of the promoter analyzed: 7 nM (Table 1); this result is significantly less than the norm: 5 nM (*Z*-score = 7.63, *α* < 10^−6^). According to some studies [39–41], this significant decrease in TBP affinity for the* IL1B* promoter corresponds to an interleukin 1*β* deficiency in patients. Because the known SNP marker rs1143627 and the unannotated SNP rs549858786 have opposite effects (relative to each other) on* IL1B* expression, we performed an additional keyword search for [54, 63] “interleukin 1*β* deficiency” as a biochemical marker relevant to medicine in the NCBI databases [4]. The result is shown in Table 1 and represents experimental findings [76] in a murine model of human rheumatoid arthritis, which showed an association of the interleukin 1*β* deficiency with a high risk of this autoimmune disease. Within the framework of this animal model of the human disease [76], we propose rs549858786 as a candidate SNP marker of an increased risk of rheumatoid arthritis. This is the first novel finding in the present study.

Furthermore, the* IL1B* promoter under study contains one more unannotated SNP rs4986962 (−67G→T) [3, 4] that was predicted by our Web service [62] to insignificantly change TBP affinity for this promoter (data not shown). Notably, this prediction of (1)–(4) and Algorithm 1 does not rule out the possible usefulness of this SNP for clinical practice as a valid SNP marker of some human diseases. This is because our prediction does not take into account the influence of this SNP, for example, on the DNA sites binding to other transcription factors [23, 77], which can be studied in a different project, for example, using other Web services [25–27].

As one can see in Table 1, the next known SNP marker (of myocardial infarction and venous thromboembolism), rs563763767 (−21C→T) [78], is located within the core-promoter for transcript number 1 of the* F3* gene (coagulation factor F3; synonym: tissue factor) and has properties that are similar to those of the above-mentioned basic example. Using the Web service [62], we predicted the SNP-caused overexpression of this gene, in agreement with the known pathogenesis of these cardiovascular diseases [78]. In turn, the known SNP marker −51T→C within the core-promoter of the human* NOS2* gene (inducible nitric oxide synthase 2) exemplifies the so-called balanced SNPs, which can have both beneficial (malaria resistance [79]) and adverse effects (epilepsy risk [80]) on human health. Another type of manifestations of SNPs is illustrated by the known SNP marker rs10168 (−26G→A) in the human* DHFR* gene (dihydrofolate reductase; the main target of methotrexate, which is the key drug for the treatment of children with acute lymphoblastic leukemia) [81]. This gene's overexpression as a result of −26A causes resistance to the above-mentioned antitumor drug.

The known SNP marker rs10895068 of the human* PGR* gene exemplifies the SNP-caused* de novo* appearance of a spurious TBP-biding site along with the additional pathogenic TSS at position +270 from the normal TSS for transcript number 2 of the same gene [82]. This alternative TSS disrupts the balance between the *α* and *β* isoforms of the progesterone receptor encoded by this gene; this aberration doubles the risk of endometrial cancer in overweight women [82].

Finally, the two bottom lines of Table 1 show two examples of the known SNP markers of so-called silent SNPs: −20A→T within the promoter of the human* CYP21A2* gene [83] and rs111426889, which precedes the alternative TSS located at position −120 upstream of the major TSS for transcript number 3 of the* TNFRSF18* gene [84]. These silent SNPs are useful for monitoring of migration flows and ethnic composition of regional human subpopulations.

### 3.2. The Results on 22 Known Biomedical SNP Markers That Decrease TBP Affinity for Human Gene Promoters

The results on 22 known biomedical SNP markers that decrease TBP affinity for human gene promoters are presented in Table 2. Let us analyze them briefly referring to the above examples.

Some of these biomedical SNP markers (8 of 22; 36%) were found within the promoters of two gene-paralogs:* HBB* and* HBD* of *β*- and *δ*-hemoglobins. As one can see in Table 2, all of them are “balanced SNPs” causing both resistance to malaria and thalassemia [85–96] with only one exception: substitution −27A→T is of the “silent SNP” type. In addition, the SNP marker rs2814778 within the* DARC* gene is of the same “balanced SNP” type; namely, it is associated with malaria resistance and a low white-blood-cell count, as positive and negative effects on human health, respectively [97].

The known SNP marker rs28399433 (low risk of lung cancer among smokers) was found here within the human* CYP2A6* gene (nicotine oxidase; synonyms: xenobiotic monooxygenase, polypeptide 6 of subfamily A of family 2 of cytochrome p450) [98, 99]. Our Web service [62] predicts (see (1)–(4) and Algorithm 1) reduced affinity of TBP for the minor allele of this gene promoter (Table 2). This result is consistent with empirical studies involving bioluminescence [98, 99]. In addition, three known SNP markers, rs55999272 in the* CRYGEP* gene, rs2276109 in* MMP12*, and 18 bp deletion within the promoter of* CETP,* are associated with a reduced risk of Coppock-like cataract [100], asthma [101], systemic sclerosis [102], psoriasis [103], and atherosclerosis [104, 105] due to the SNP-caused damage to the TBP-binding sites of the promoters of these genes.

In addition, the known SNP marker rs34223104 within the core-promoter for the undocumented alternative TSS (located 48 bp upstream of the major TSS of the CYP2B6 gene) transforms the canonical form (TATA-box) of the TBP-binding site, 5′-gatgaaatttTATAAcagggt-3′, into the C∖EBP-binding site (C∖EBP, CCAAT-enhancer-binding protein), which causes increased bioactivation of the anticancer prodrug cyclophosphamide [106]. In this case, our Web service [62] predicts damage to this normal TBP-binding site that is in agreement within the experimentally observed transformation of this TBP-binding site into the SNP-caused C∖EBP-binding site [106].

Furthermore, the remaining six known SNP markers, rs7277748 (*SOD1*) [107], rs1800202 (*TPI1*) [108, 109], rs35036378 (*ESR2*) [110, 111], rs201739205 (*HSD17B1*) [112], rs72661131(*MBL2*) [113–115], and rs17537595 (*ADH7*) [116], including two substitutions, −35A→C (*APOA1*) [117] and −33A→C (F7) [118], are of the most frequent and best understood type of SNP: pathogenic damage to a normal TBP-binding site. This way, these SNPs can reduce expression of human genes.

Finally, near these 22 known biomedical SNP markers, we found and proposed 13 candidate SNP markers: rs63750953 (*HBB*), rs281864525 (*HBB*), rs34166473 (*HBD*), rs55878706 (*DARC*), rs572527200 (*MMP12*), rs17231520 (*CETP*), rs569033466 (*CETP*), rs563558831 (*CYP2B6*), rs562962093 (*MBL2*), rs72661131 (*MBL2*), rs372329931 (*ADH7*), rs36773297 (*F7*), and rs549591993 (*F7*), as one can see in Table 2. About a half of them (8 of 13, 62%) have effects on gene expression that are codirectional with the effects of the nearby known SNP markers and thus can serve as markers of the same human diseases (e.g., rs562962093 and rs33931746). For the other half of the SNPs, we found associations with appropriate diseases [119, 120] using a keyword search [54, 63] in NCBI databases [4] (e.g., rs567653539).

### 3.3. The Results on 10 Known Biomedical SNP Markers That Insignificantly Change TBP Affinity for Human Gene Promoters

The results on 10 known biomedical SNP markers that insignificantly change TBP affinity for human gene promoters are presented in Table 3. Let us discuss them briefly.

First of all, the known SNP marker rs1394205 (−29G→A) within the* FSHR* gene belongs to one of the most important types of SNP: it causes a frequently occurring disease, for example, male infertility, and this connection has been proven clinically regardless of bioinformatic, biochemical, or any other nonclinical data. As shown in the first line of Table 3, in terms of this biomedical marker, there are no differences between fertile men (who are fathers) and infertile men in Italy [121] and in Turkey [122]. In agreement with these biomedical findings [121, 122], our Web service [62] (see (1)–(4) and Algorithm 1) predicts no differences in TBP affinity for this gene's promoter between ancestral and minor alleles of this SNP.

The next four substitutions, −48G→C (*F9*), −42T→A (*F9*), rs16887226 (*StAR*), and rs28399433 (*GH1*), are among the oldest known SNP markers that were discovered by means of the electrophoretic mobility shift assay (EMSA) before the advent of the reference human genome, gh19 [123, 124, 126]. According to these EMSA assays [123, 124, 126], each of these four SNPs pathologically reduces expression of the corresponding gene by disrupting the tissue-specific binding site for a transcription factor rather than by disrupting the ubiquitous TBP-binding site (they overlap). Additionally, the next five known SNP markers—rs1332018 (*GSTM3*), rs7586110 (*UGT1A7*), rs10465885 (GJA5), rs35594137 (GJA5), and rs13306848 (*THBD*)—have properties similar to those of the SNPs above, in terms of bioluminescence (LUC) assays [127–132] instead of EMSA. Here we found six nearby unannotated SNPs, rs371045754 (*F9*), rs544850971 (*StAR*), rs200209906 (*GSTM3*), rs574890114 (*UGT1A7*), rs542729995 (*UGT1A7*), and rs587745372 (*GJA5*), which can significantly disrupt the above-mentioned TBP-binding sites and thereby may cause the same diseases in humans as do the six candidate SNP markers (Table 3).

Finally, the last two biomedical SNP markers—rs587745372 and rs398048306—taken together are the well-known unique genetic variation in the TBP-binding site length, A (TA)_5–8_A in comparison with the norm: A (TA)_7_A. The longest of them, rs587745372, is an integral part of several haplotypes associated with a high risk of hyperbilirubinemia and jaundice [133], whereas two shortest ones, rs398048306 and rs200209906, are “silent SNPs” that are used to study ethnic differences of regional human subpopulations ([12] and Table 3).

Thus, in the vicinity of the 40 known biomedical SNP markers within the TBP-binding sites in humans, we first found 17 candidate SNP markers: rs55878706 (malaria resistance, low white-blood-cell count), rs562962093 (stroke, preeclampsia, and variable immunodeficiency), rs563558831 (cyclophosphamide bioactivation), rs549858786 (rheumatoid arthritis), rs372329931 (esophageal cancer), rs72661131 (cardiovascular events in rheumatoid arthritis), rs200209906 (brain, lung, testicular, and renal cell carcinomas), rs572527200 (low risk of asthma, systemic sclerosis, and psoriasis), rs371045754 (Leiden hemophilia B), rs587745372 (cardiovascular problems), rs367732974 and rs549591993 (both: progression of colorectal cancer from a primary tumor to metastasis), rs17231520 and rs569033466 (both: atherosclerosis), and rs63750953, rs281864525, and rs34166473 (all three: malaria resistance, thalassemia). This is the main result of our study.

## 4. Discussion

Because the mainstream method of searching for candidate SNP markers is now based on a statistical estimate of the similarity between the projections of unannotated SNPs and known SNP markers on various genome-wide maps, here we simplified the procedure by limiting it to unannotated SNPs only that are located near the known SNP markers in the TBP-binding sites of human genes. Within this framework, we found and analyzed 40 known SNP markers and 163 nearby unannotated SNPs shown within the first column of Tables 1–3 below the gene acronyms. The majority of the unannotated SNPs (153 of 203; 75%) appear to be insignificantly altering TBP affinity for the core-promoter of the corresponding gene in humans (data not shown). This prediction of our Web service [62] seems to be consistent with the commonly accepted paradigm of genetic stability of the human genome and with data from EMSA and LUC assays of SNP-caused pathological disruption of binding sites for tissue-specific transcription factors rather than disruption of the TBP-binding site (overlaps them; they constitute the so-called composite unit [134]; Table 3).

The second most frequent group of SNP markers, 37 of 203 (18%), disrupts TBP-binding sites within core-promoters of human genes and thereby reduces expression of these genes; this deficient gene expression is more often associated with adverse than beneficial effects on human health. This finding is in agreement with the commonly accepted bioinformatics notion that the SNP-caused damage to genetic information is more frequent than SNP-caused genetic benefits.

The third most frequent group of SNP markers, 13 of 203 (7%), increases the TBP binding affinity for core-promoters of human genes and, hence, causes overexpression of these genes. This overexpression can be pathogenic, neutral, or beneficial for human health at approximately equal probabilities. This finding points to huge diversity of genetic effects of SNPs within the human genome. Indeed, the remaining manifestations of SNPs constitute only rare examples, such as “silent SNPs” (e.g., rs111426889), “balanced SNPs” (e.g., rs35518301), a* de novo* occurrence of a spurious TBP-biding site (e.g., rs10895068), transformation of a normal TBP-binding site into another regulatory genomic signal (e.g., rs34223104), a change of the composite unit containing the TBP-binding site (e.g., rs28399433), a deletion of the DNA fragment either around or inside the TBP-binding site (e.g., rs63750953), and a duplication of the DNA fragment inside the TBP-binding site (e.g., rs34983651).

As for the SNP-caused pathological changes, the majority (40 of 57; 70%) of the SNP markers of diseases are either increasing or decreasing the risk of human diseases, whereas the rare types of SNPs are associated with drug resistance (e.g., rs10168), prodrug bioactivation (e.g., rs34223104), disease complications (e.g., rs72661131), and ethnic differences (e.g., rs398048306 and rs34223104). In addition, 10 of the 17 proposed candidate SNP markers are codirectionally changing TBP affinity for the core-promoters of human genes with respect to the nearby known SNP markers, whereas the remaining 7 candidate SNP markers do so in the opposite direction. Accordingly, we did additional keyword searches [54, 63] by hand in NCBI databases [4]. Both of these observations mean that our Web service [62], when combined with a manual comprehensive search for keywords [54, 63] by means of the Web-based information sources, is most suitable for precise analysis of specific SNPs, genes, and diseases rather than for a whole-genome search for a wide range of all possible manifestations of any unannotated SNPs.

In this regard, it should be noted that the statistical significance of the proposed 17 candidate SNP markers varies from high confidence (*α* < 10^−7^) to borderline significance (*α* < 0.05). In contrast, *K*_*D*_ values when expressed in moles (*M*; representing affinity of TBP binding to the core-promoter* in vitro* [50]) vary from 1 nM to 62 nM, and their variation among alleles of a given SNP is less than 2% of this range and thus outside the limits of accuracy of empirical measurement of *K*_*D*_ values, if we are not taking into account additional information on the expected range of the values being measured. Thus, the *K*_*D*_ values shown in Tables 1–3 are necessary for prognostic affinity analysis of these 17 candidate SNP markers that we made using the Web service [62] for the purpose of their empirical verification by means of sophisticated equipment (e.g., [50–53]).

Finally, our estimates for the 17 candidate SNP markers (Tables 1–3) are only measures of bioinformatic (*K*_*D*_-values, *Z*-score, *α*-value, *p* value, etc.) and biomedical justification (last columns in Tables 1–3) for the highly expensive and laborious verification of SNPs during a search for an SNP marker that can be validated only by a higher incidence in patients than in healthy people. What is healthy or normal depends on ethnic, social, age, and gender composition of a human subpopulation, the settlement ratio and the associated migration flows, climate and environment, living conditions and lifestyle, the technological level of health care and diagnostic procedures, anamnesis, and treatment history [135].

## 5. Conclusions

The use of biomedical SNP markers can improve effectiveness of treatment and help to develop new medications. The majority of known SNP markers are located in protein-coding regions of human genes and have invariant manifestation of disruption in the protein structure and/or function (e.g., [29]). At the same time, only a minority of known SNP markers are located in regulatory regions of genes because their experimental detection is complicated by the tissue- and developmental-stage-specific variation in binding of a regulatory protein to the these DNA regions [23, 25, 27, 30, 77]. Nevertheless, the best-studied regulatory SNPs in TBP-binding sites of human promoters seem to have a lot in common with the SNPs in protein-coding regions rather than with the remaining regulatory SNPs. With this in mind, here we first predicted 17 candidate biomedical SNP markers in TBP-binding sites of human promoters and confirmed them using both clinical and basic research of other investigators (Tables 1–3). Verification of these predictions according to established biomedical standards and protocols can bridge the gap between the best-studied SNPs within protein-coding regions of human genes and the worst-studied regulatory SNPs and thus may advance postgenomic predictive preventive personalized medicine.

## Figures and Tables

**Algorithm 1:**
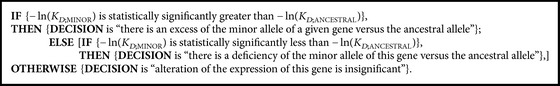


**Table 1:** Known disease-related SNP markers increasing affinity of the TATA-binding protein (TBP) for human gene promoters, their SNP neighbors.

Gene (*N*_SNP_)	RNA (TSS)	dbSNP [4] rel. 141, 142	SNP hg19→min	5′-flank	**hg19** *min*	3′-flank	min versus hg19 *K*_*D*_, nM	Δ	*Z*	*α*	Known [reference] diseases or *hypothetical [this work] ones*	[Reference], [this work]
*IL1B* (3)	#2 (+1)	rs1143627	−31c→t	ttttgaaagc	**c** *t*	ataaaaacag	2 versus 5	↑	15	10^−7^	Gastric cancer in *Helicobacter pylori* infection, hepatocellular carcinoma in hepatitis C virus infection, non-small cell lung cancer, chronic gastritis and gastric ulcer in* H. pylori* infection, Graves' disease, and major recurrent depression	[10, 70–75]
		*rs549858786*	−28a→t	tgaaagccat	**a** *t*	aaaacagcga	*7 *versus *5*	↓	*8*	*10* ^−7^	*(Hypothetically)* *Rheumatoid arthritis*	[This work], [76]

*F3* (2)	#1 (+1)	rs563763767	−21c→t	ccctttatag	**c** *t*	gcgcggggca	2 versus 3	↑	6	10^−7^	Myocardial infarction and venous thromboembolism	[78]

*NOS2* (7)	#1 (+1)	ND, see [79]	−51t→c	gtataaatac	**t** *c*	tcttggctgc	1 versus 2	↑	3	10^−2^	Resistance to malaria, epilepsy risk	[79, 80]

*DHFR* (5)	#3 (+1)	rs10168	−26g→a	ctgcacaaat	**g** *a*	gggacgaggg	9 versus 15	↑	9	10^−7^	Resistance to methotrexate therapy for leukemia	[81]

*PGR* (3)	#2 (+270)	rs10895068	−26g→a	gggagataaa	**g** *a*	gagccgcgtg	6 versus 10	↑	8	10^−7^	Endometrial cancer caused by a *de novo* occurrence of a spurious TBP-biding site	[82]
		*rs544843047*	*−33t→c*	*agtcgggaga*	***t*** *c*	*aaaggagccg*	*22 *versus *10*	↓	*14*	*10* ^−7^	*(Hypothetically)* *Health*	[This work]

*CYP21A2* (1)	#2 (+1)	ND, see [83]	−20a→t	gtcattccag	**a** *t*	aaagggccac	13 versus 24	↑	9	10^−7^	A healthy Hungarian blood donor participating in a health check-up program	[83]

*TNFRSF18* (5)	#3 (−120)	rs111426889	−25c→t	gtgctataaa	**c** *t*	gccgccccct	2 versus 4	↑	8	10^−7^	A healthy individual in the “Control” cohort selected for comparison with the “Autoimmune Diseases” cohort	[84]

Note: *N*_SNP_, total number of SNPs processed; RNA, item number of mRNA in GENCODE v.19 [6]; TSS, transcription start site; hg19, ancestral allele; min, minor allele; *K*_*D*_, an estimate [55] of the dissociation constant (*K*_*D*_) of the TBP-DNA complex *in vitro* [50]; ND, not documented; Δ, the expression change in comparison with the norm: overexpression (↑), deficient expression (↓), and norm (=); *Z*, *Z*-score; *α* = 1 − *p*, significance (*p*, probability; Figure 1); TF, transcription factor; EMSA, electrophoretic mobility shift assay; CAT, chloramphenicol acetyl transferase activity; LUC, bioluminescence.

**Table 2:** Known disease-related SNP markers decreasing affinity of the TATA-binding protein (TBP) for human gene promoters, their SNP neighbors.

Gene (*N*_SNP_)	RNA (TSS)	dbSNP [4] rel. 141, 142	SNP hg19→min	5′-flank	**hg19** *min*	3′-flank	min versus hg19 *K*_*D*_, nM	Δ	*Z*	*α*	Known [reference] or hypothetical [this work] diseases (observations)	[Reference], [this work]
*HBB* (19)	#2 (+1)	rs397509430	del-29t	gggctgggca	**t** —	atacaacagt	29 versus 5	↓	34	10^−7^	Malaria resistance and *β*-thalassemia	[85–92]
		rs33980857	−29t→a,g,c	gggctgggca	**t** *a,g,c*	atacaacagt	21 versus 5	↓	27	10^−7^		
		rs34598529	−28a→g	ggctgggcat	**a** *g*	aaagtcaggg	18 versus 5	↓	24	10^−7^		
		rs33931746	−27a→g,c	gctgggcata	**a** *g,c*	aagtcagggc	11 versus 5	↓	14	10^−7^		
		rs33981098	−30a→g,c	agggctgggc	**a** *g,c*	taaaagtcag	9 versus 5	↓	10	10^−7^		
		rs34500389	−31c→a,t,g	cagggctggg	**c** *a,t,g*	ataaaagtca	6 versus 5	↓	3	10^−2^		
		ND, see [93]	−27a→t	gctgggcata	**a** *t*	aagtcagggc	3 versus 5	↑	8	10^−2^	Health, well-known so-called “silent SNP”	[93, 94]
		*rs63750953*	*del*-*25aa*	*ctgggcataa*	***aa*** —	*gtcagggcag*	*8 *versus *5*	↓	*9*	*10* ^−7^	*(Hypothetically) Malaria resistance, β-thalassemia*	[This work], [95]
		*rs281864525*	*−25a→c*	*tgggcataaa*	***a*** *c*	*gtcagggcag*	*7 *versus *5*	↓	*7*	*10* ^−7^		

*HBD* (14)	#3 (+1)	rs35518301	−31a→g	caggaccagc	**a** *g*	taaaaggcag	8 versus 4	↓	11	10^−7^	Malaria resistance and *δ*-thalassemia	[9, 96]
		*rs34166473*	*−30t→c*	*aggaccagca*	***t*** *c*	*aaaaggcagg*	*8 *versus *4*	↓	*18*	*10* ^−7^	*(Hypothetically) Malaria resistance, δ-thalassemia*	[This work], [95]

*DARC* (2)	#3 (+1)	rs2814778	−26t→c	ttggctctta	**t** *c*	cttggaagca	12 versus 10	↓	4	10^−3^	Low white-blood-cell count and resistance to malaria	[9, 97]
		*rs55878706*	*−27a→t(c)*	*cttggctctt*	***a*** *t(c)*	*tcttggaagc*	*12 *versus *10*	↓	*4*	*10* ^−3^	*(Hypothetically) Low white-blood-cell count and malaria resistance *	[This work]

*CYP2A6* (3)	#3 (+1)	rs28399433	−34t→g	tcaggcagta	**t** *g*	aaaggcaaac	9 versus 2	↓	21	10^−7^	Lower risk of lung cancer in smokers LUC: “−34*g*” has 50% of “−34**t**”	[98, 99]

*CRYGEP* (5)	#1 (−140)	rs55999272	−28t→c	tcctgctata	**t** *c*	agccccgccg	5 versus 2	↓	11	10^−7^	For “−28**t**” ancestral allele (norm), risk of Coppock-like cataract	[100]

*MMP12* (2)	#1 (+1)	rs2276109	−27a→g	gatatcaact	**a** *g*	tgagtcactc	14 versus 11	↓	3	10^−2^	Low risk of chronic asthma, systemic sclerosis, and psoriasis	[101–103]
		*rs572527200*	*−30a→g*	*gatgatatca*	***a*** *g*	*ctatgagtca*	*14 *versus *11*	↓	*3*	*10* ^−2^	*(Hypothetically) Low risk of asthma, systemic sclerosis, *and* psoriasis*	[This work]

*CETP* (5)	#4 (+1)	ND, see [104]	del-54 [18 bp]	cgtgggggct	**[18 bp]** —	gggctccagg	7 versus 4	↓	7	10^−7^	Hyperalphalipoproteinemia reduces atherosclerosis risk	[104, 105]
		*rs17231520*	*−68g→a*	*ggggctgggc*	***g*** *a*	*gacatacata*	*2 *versus *4*	*↑*	*10*	*10* ^−7^	*(Hypothetically) Higher risk of atherosclerosis-related autoimmune diseases*	[This work], [105]
		*rs569033466*	*−53g→a*	*atacatatac*	***g*** *a*	*ggctccaggc*	*3 *versus *4*	*↑*	*4*	*10* ^−3^		

*CYP2B6* (4)	#1 (−48)	rs34223104	−28t→c	gatgaaattt	**t** *c*	ataacagggt	10 versus 4	↓	15	10^−7^	Better bioactivation of anticancer prodrug cyclophosphamide	[106]
		*rs563558831*	*−26t→c*	*tgaaatttta*	***t*** *c*	*aacagggtgc*	*10 *versus *4*	↓	*13*	*10* ^−7^	*(Hypothetically) Better bioactivation of cyclophosphamide*	[This work]

*SOD1* (4)	#4 (+1)	rs7277748	−32a→g	ggtctggcct	**a** *g*	taaagtagtc	7 versus 2	↓	17	10^−7^	Familial amyotrophic lateral sclerosis	[107]

*TPI1* (3)	#201 (+1)	rs1800202	−24t→g	gcgctctata	**t** *g*	aagtgggcag	4 versus 1	↓	17	10^−7^	Hemolytic anemia and neuromuscular diseases	[108, 109]

*ESR2* (5)	#1 (+1)	rs35036378	−43t→g	cctctcggtc	**t** *g*	ttaaaaggaa	8 versus 6	↓	5	10^−3^	ESR2-low pT1 tumor	[110, 111]

*HSD17B1* (8)	#2 (+1)	rs201739205	−36a→c	aggtgatatc	**a** *c*	agcccagagc	18 versus 13	↓	5	10^−3^	Breast cancer	[112]

*MBL2* (6)	#1 (+1)	rs72661131	−39t→c	tctatttcta	**t** *c*	atagcctgca	4 versus 2	↓	12	10^−7^	Variable immunodeficiency, stroke, and preeclampsia	[113–115]
		*rs562962093*	*−40a→g*	*atctatttct*	***a*** *g*	*tatagcctgc*	*5 *versus *2*	↓	*15*	*10* ^−7^	*(Hypothetically) Stroke, variable immunodeficiency, *and* preeclampsia*	[This work]
		*rs72661131*	*−35g→a*	*tttctatata*	***g*** *a*	*cctgcaccca*	*1 *versus *2*	*↑*	*12*	*10* ^−7^	*(Hypothetically) Risk of cardiovascular events in rheumatoid arthritis*	[This work], [119]

*ADH7* (3)	#3 (+1)	rs17537595	−36t→c	gctgctgtta	**t** *c*	atacaacaga	3 versus 1	↓	13	10^−7^	Esophageal cancer	[116]
		*rs372329931*	*−37a→g*	*agctgctgtt*	***a*** *g*	*tatacaacag*	*3 *versus *1*	↓	*13*	*10* ^−7^	*(Hypothetically) Esophageal cancer*	[This work]

*APOA1* (1)	#3 (+1)	ND, see [117]	−35a→c	tgcagacata	**a** *c*	ataggccctg	4 versus 3	↓	5	10^−3^	Hematuria, fatty liver, obesity	[117]

*F7* (4)	#1 (+1)	ND, see [118]	−33a→c	ccttggaggc	**a** *c*	gagaactttg	62 versus 53	↓	3	10^−2^	Moderate bleeding tendency	[118]
		*rs367732974*	*−19g→a*	*aactttgccc*	***g*** *a*	*tcagtcccat*	*47 *versus *53*	*↑*	*2*	*0.05*	*(Hypothetically) Risk of progression of colorectal cancer from a primary tumor to metastasis*	[This work], [120]
		*rs549591993*	*−13c→a*	*gcccgtcagt*	***c*** *a*	*ccatggggaa*	*25 *versus *53*	*↑*	*13*	*10* ^−7^		

Note: hereinafter, can be seen under Table 1.

**Table 3:** Known disease-related SNP markers insignificantly changing TBP affinity for human gene promoters, their SNP neighbors.

Gene (*N*_SNP_)	RNA (TSS)	dbSNP [4]	SNP hg19→min	5′-flank	**hg19** *min*	3′-flank	min versus hg19 *K*_*D*_, nM	Δ	*Z*	*α*	Known [reference] or hypothetical [this work] diseases	[Reference], [this work]
*FSHR* (3)	#2 (+16)	rs1394205	−29g→a	gcaaatgcag	**g** *a*	aagaaatcag	7.3 versus 7.3	=	0	>0.05	No differences between proven fathers and infertile men	[121, 122]

*F9* (4)	#1 (+1)	ND, see [123]	−48g→c	agctcagctt	**g** *c*	tactttggta	6.4 versus 6.4	=	0	>0.05	Leiden hemophilia B, EMSA: HNF4-binding site disrupted rather than proximal TBP-binding site	[123]
		ND, see [123]	−42t→a	gcttgtactt	**t** *a*	ggtacaacta	6.4 versus 6.4	=	0	>0.05		
		*rs371045754*	*−32a→c*	*tggtacaact*	***a*** *c*	*atcgacctta*	*9.6 *versus* 6.4*	↓	*5*	*10* ^−7^	*(Hypothetically)* *Leiden hemophilia B*	[This work]

*StAR* (3)	#3 (+31)	rs16887226	−33c→t	cagccttcag	**c** *t*	gggggacatt	10.3 versus 10.3	=	0	>0.05	Hypertensive diabetic patients, EMSA: unknown TF-binding site disrupted rather than TBP-binding site	[124]
		*rs544850971*	*−22a→g*	*tcagcggggg*	***a*** *g*	*catttaagac*	*12.1 *versus* 10.3*	↓	*5*	*10* ^−2^	*(Hypothetically)* *Congenital adrenal hyperplasia*	[This work], [125]

*GH1* (11)	#1 (+1)	rs28399433	del-50g	aggggccagg	**g** *—*	tataaaaagg	1.4 versus 1.5	=	1	>0.05	Short stature, EMSA: unknown TF-binding site disrupted rather than TBP-binding site	[126]

*GSTM3* (8)	#4 (+1)	rs1332018	−49c→a	ccccttatgt	**c** *a*	gggtataaag	3.1 versus 3.6	=	1.9	>0.05	Risk of brain, lung, testicular, and renal cell carcinomas, LUC: “−49**c**” is 10% of “−49*a*”	[127, 128]
		*rs200209906*	*−36c→t,a*	*gtataaagcc*	***c*** *t,a*	*ctcccgctca*	*4.3 *versus* 3.6*	↓	*2.4*	*<0.05*	*(Hypothetically) Risk of brain, lung, testicular, and renal cell carcinomas*	[This work]

*UGT1A7* (4)	#1 (+1)	rs7586110	−57t→g	cttcttccac	**t** *g*	tactatatta	1.48 versus 1.54	=	1	>0.05	Oral cancer risk, LUC: “−57*g*” is 50% of “−57**t**”	[129]
		*rs574890114*	*−55a→g*	*tcttccactt*	***a*** *g*	*ctatattata*	*2.02 *versus* 1.54*	↓	*4*	*10* ^−3^	*(Hypothetically)* *Higher risk of oral cancer*	[This work]
		*rs542729995*	*−52a→g*	*tccacttact*	***a*** *g*	*tattatagga*	*2.28 *versus* 1.54*	↓	*5*	*10* ^−7^		

*GJA5* (8)	#1 (+1)	rs10465885	−55g→a	caactaagat	**g** *a*	tattaaacac	3.1 versus 3.4	=	1	>0.05	Arrhythmia, cardiovascular events LUC: “−55**g**” is 50% of “−55*a*”	[130]
	#2 (+1)	rs35594137	−39g→a	gaggagggaa	**g** *a*	gcgacagata	5.7 versus 5.7	=	0	>0.05	Arrhythmia, cardiovascular events LUC: “−39*a*/76g” is 50% of “**−39g/76a**”	[131]
		*rs587745372*	*−29a→t*	*ggcgacagat*	***a*** *t*	*cgattaaaaa*	*6.8 *versus* 5.7*	↓	*3*	*10* ^−3^	*(Hypothetically)* *Arrhythmia, cardiovascular events*	[This work]

*THBD* (3)	#1 (+68)	rs13306848	−33g→a	agggagggcc	**g** *a*	ggcacttata	2.3 versus 2.1	=	1	10^−7^	Thrombophlebitis risk LUC: “−33*a*” is 84% of “−33**g**”	[132]

*UGT1A1* (10)	#201 (+1)	rs34983651	ins-55at	ggtttttgcc	**—** *at*	atatatatat	0.65 versus 0.67	=	1	>0.05	Necessary but not sufficient in hyperbilirubinemia and jaundice	[133]
		rs398048306	del-51(at)_1;2_	ggtttttgcc	**at(at)** *—*	atatatatat	0.71 versus 0.67	=	1	>0.05	Ethnic differences such as rare alleles in humans	[12]
